# Impact of Chronic Total Occlusion in a Noninfarct-related Artery on Clinical Outcomes in Patients With Acute ST-elevation Myocardial Infarction Undergoing Primary Percutaneous Coronary Intervention

**DOI:** 10.1097/MD.0000000000002441

**Published:** 2016-01-15

**Authors:** Hui-Ping Zhang, Ying Zhao, Hui Li, Guo-Dong Tang, Hu Ai, Nai-Xin Zheng, Jing-Hua Liu, Fu-Cheng Sun

**Affiliations:** From the Department of Cardiology, Beijing Hospital, The Fifth Affiliated Hospital of Peking University (H-PZ, YZ, HL, G-DT, HA, N-XZ, F-CS); and Department of Cardiology, Beijing Anzhen Hospital, Capital Medical University, Beijing Institute of Heart Lung and Blood Vessel Diseases, Beijing, China (J-HL).

## Abstract

In the setting of primary percutaneous coronary intervention (PCI), encountering with chronic total occlusion (CTO) in a noninfarct-related artery (IRA) is not a rare situation. Limited information on the impact of CTO on clinical outcomes in acute ST-elevation myocardial infarction (STEMI) patients undergoing primary PCI has raised more concerns. The aim of the present study was to evaluate the effect of concurrent CTO in a non-IRA on the clinical outcomes in patients with STEMI undergoing primary PCI.

In the present prospective study, 555 consecutive patients with STEMI who underwent early primary PCI from January 2010 to December 2013 were included. The patients were divided into 2 groups: no CTO and CTO. Data on 12 months follow-up was obtained from 449 patients. The primary endpoint was the composite of hospitalization from angina, reinfarction, heart failure, or re-revascularization, and cardiac death at 12 months follow-up.

Of the 555 patients, 75 (13.5%) had CTO in a non-IRA. Compared with patients in no CTO group, more patients in CTO group had hypertension (62.7% vs 46.5%, *P* = 0.009), diabetes (49.3% vs 35.0%, *P* = 0.024), and 3-vessel disease (52.0% vs 32.3%, *P* = 0.001). Patients with CTO had a lower left ventricular ejection fraction (LVEF) (40.1% ± 16.8% vs 54.3% ± 12.1%, *P* = 0.038), more presented with cardiogenic shock on admission (13.3% vs 4.8%, *P* = 0.008), compared with patients without CTO. Complete revascularization (CR) was less achieved in CTO group than in no CTO group (33.3% vs 49.1%, *P* = 0.013). The 12-month cardiac mortality rate was 14.5% versus 6.2% (*P* = 0.039), the incidence of 12-month primary endpoint was 38.7% versus 21.2% (*P* = 0.003) for CTO and no CTO group, respectively. Multivariate analysis revealed that after correction for baseline differences, CTO in a non-IRA (hazard ratio 4.183, 95% confidence interval 1.940–6.019, *P* = 0.001), cardiogenic shock on admission (hazard ratio 3.286, 95% confidence interval 1.097–9.845, *P* = 0.034), and 3-vessel disease (hazard ratio 2.678, 95% confidence interval 1.221–5.874, *P* = 0.014) remained an independent predictor of 1-year cardiac mortality in patients with STEMI undergoing primary PCI.

CTO in a non-IRA in patients with STEMI undergoing primary PCI is associated with a poor prognosis. The presence of CTO in a non-IRA, cardiogenic shock on admission and 3-vessel disease might be an independent risk factor for greater 1-year cardiac mortality in patients with acute STEMI undergoing primary PCI.

## INTRODUCTION

Data from registries and randomized trials show that an early strategy of primary percutaneous coronary intervention (PCI) is the effective way to recanalize infarct-related artery (IRA) and improve clinical outcomes in patients with acute ST-elevation myocardial infarction (STEMI).^1,2^ In the setting of primary PCI for acute STEMI, encountering with chronic total occlusion (CTO) in a non-IRA is not a rare situation. Among patients with acute STEMI who have a clinical indication for primary PCI, the incidence of CTO in a non-IRA has been reported to be 12% to 13%.^3,4^ Generally, CTO reflects the severity of the diseased coronary vessels, thus the presence of CTO in a non-IRA usually makes primary PCI performed more discreetly, more clinical consideration may be needed.^5,6^

Although IRAs can often succeed being opened in emergent setting, it was supposed that the rate of complete revascularization (CR) in acute STEMI patients with CTO was lower than STEMI patients without CTO.^7,8^ So far, there is still a paucity of data relating to impact of CTO on clinical outcomes in acute STMI patients undergoing primary PCI. We conducted a prospective single-center analysis of patients with acute STEMI undergoing primary PCI to identify the differences in clinical characteristics and outcomes in patients with and without CTO. Factors including concurrent CTO in a non-IRA that might affect the long-term prognosis were evaluated.

## METHODS

### Study Population and Clinical Data

The study was approved by the institutional Ethics Committee, all patients signed informed consent to undergo coronary angiography and intervention procedure. All patients were informed of the study plan, individual informed consent was waived given the institutional ethics regulations with regard to observational research.

All consecutive patients who underwent primary PCI for STEMI from January 2010 to December 2013 were enrolled in a prospective registry. STEMI was diagnosed when patients had symptoms of myocardial ischemia in association with persistent electrocardiographic (ECG) ST elevation >1 mm (0.1 mv) in ≧2 contiguous leads, and subsequent release of biomarkers of myocardial necrosis.^9^ New or presumably new left bundle-branch block has been considered an STEMI equivalent. Cardiogenic shock was defined clinically as symptoms of shock or peripheral hypoperfusion and hemodynamically as systemic systolic pressure <90 mmHg or systemic systolic pressure 90 to 110 mmHg while using inotropic drugs. Primary PCI was administered to eligible patients with STEMI with symptom onset within the prior 12 hours. According to whether there being a CTO lesion in a non-IRA or not, patients were classified as with CTO in non-IRA (CTO group) and without CTO in non-IRA (no CTO group). Clinical data including age, gender, current smoker, previous myocardial infarction, blood pressure (BP), serum low-density lipoprotein cholesterol (LDL-C), and creatinine (Cr) levels were recorded on admission. Estimated glomerular filtration rate (eGFR) was calculated according to the equation of MDRD.^10^ Hypertension, diabetes were recorded as comorbid conditions. Left ventricular ejection fraction (LVEF) was obtained by ultrasound cardiogram after admission.

### Primary Percutaneous Coronary Intervention Procedure

Patients with STEMI who have indication for primary PCI were immediately transported to the catheterization laboratory and underwent immediate angiography with the aim of performing primary PCI. In patients complicated by cardiogenic shock on admission, depending on their clinical status, intra-aortic balloon pumping (IABP) was inserted. Procedure adjunctive therapy during the procedure included heparin with an initial bolus of 80 to 100 U/kg and additional boluses of 1000 U/hour, whereas the use of glycoprotein IIb/IIIa inhibitors and thrombosuction was at the discretion of the operator.

If the coronary anatomy was suitable for PCI, the procedures were performed. Multivessel coronary disease (MVD), including 2-vessel coronary disease and 3-vessel coronary disease, was defined as at least 75% diameter stenosis in more than 1 major epicardial coronary artery or its main branch as assessed by quantitative coronary angiographic analysis and left main disease with diameter stenosis ≥50%. CTO was defined as 100% luminal stenosis without anterograde flow in a non-IRA, whether the anterograde bridge collaterals or retrograde contralateral vessels being present or not. A possible acute myocardial infarction (AMI) history documented in the same territory, notable ECG changes and the characteristics of the occlusion (featured by the presence of collateral circulation and acute thrombus) were differentiated between acute coronary occlusion and CTO. PCI were performed utilizing standard techniques according to guidelines.^9^ Whichever balloons and stents were used depended upon the operator's decision. Instant success of primary PCI was defined as recovering of final thrombolysis in myocardial infarction (TIMI) flow grade 2 to 3 with stent implantation or not. Revascularization after primary PCI included PCI aimed at CTO or non-CTO lesions and coronary artery bypass grafting (CABG) after index procedure. CR was defined as successfully attempting all diseased (≧70% stenosis) lesions in major epicardial coronary vessels or its major branch either during the index hospitalization or at a staged procedure.^6^

## MEDICAL TREATMENT

All patients with acute STEMI were treated with 100 to 300 mg aspirin and a loading dose of 300 mg of clopidogrel before primary PCI. Aspirin 100 mg/day were prescribed indefinitely, clopidogrel 75 mg/day was continued for at least 12 months thereafter. The choices of low molecular heparin (LWMH) and fondaparinux after PCI were at the discretion of the operator according to the angiographic characteristics of culprit lesion and thrombus burden. Patients also received other medications including statins, nitrates, angiotensin converting enzyme inhibitors (ACEI), angiotensin receptor inhibitors (ARB), and β-receptor blocker (β-B), if these agents were not otherwise contraindicated.

### Clinical Follow-up

The clinical data from all patients with STEMI were prospectively recorded in a computerized database as a part of a single center acute coronary syndromes registry. Follow-up data including information on cardiac medication were obtained by direct telephone interviews and outpatient visits. All patients were scheduled for clinical follow-up at 6- and 12-month. Inconsistent data were completed by review of the hospital records and outpatient reports. The primary endpoint of the study was the composite of hospitalization from angina, reinfarction, heart failure, or re-revascularization, and cardiac death at 12-month of follow-up. All deaths were considered cardiac related unless an unequivocal noncardiac cause could be documented.

### Statistical Analysis

Continuous variables with a normal distribution are expressed as mean ± SD, while categorical data are reported as number and percentages. Unpaired Student's *t*-test was used to assess differences among continuous variables, and the *χ*^2^ test or Fisher exact test were used for comparisons of categorical data. Cumulative event curves were generated using Kaplan–Meier method, and the differences of event rates between groups were evaluated by log rank analysis. We performed multivariable analysis using a forward stepwise Cox proportional hazards regression model to evaluate the effect of particular variables on 1-year cardiac mortality, expressed as hazard ratio, with 95% confidence interval. All possible confounding factors including clinical, angiographic, and procedure variables were entered the risk-adjusted models. The c-statistic and goodness-of-fit with Hosmer and Lemeshow test were used to assess model discrimination. All *P* values were 2-sided, a *P* value < 0.05 was considered statistically significant. The software package of SPSS version 19.0 (SPSS Inc., Chicago, IL) was used for all calculations and statistical analyses.

## RESULTS

### Baseline Clinical, Angiographic, and Treatment Characteristics

The baseline clinical, angiographic, and treatment characteristics of the 555 patients are listed in Table 1. From January 2010 to December 2013, 555 consecutive patients with STEMI underwent primary PCI at our center, we found 75 (13.5%) had CTO in a non-IRA (CTO group). More patients in CTO group were current smoker (56.0% vs 42.3%, *P* = 0.026), had a greater prevalence of hypertension (62.7% vs 46.5%, *P* = 0.009), diabetes (49.3% vs 35.0%, *P* = 0.024), and cardiogenic shock on admission (13.3% vs 4.8%, *P* = 0.008) than did the patients in no CTO group. Compared with patients without CTO in a non-IRA, patients with CTO had a lower LVEF (40.1% ± 16.8% vs 54.3% ± 12.1% *P* = 0.038).

**TABLE 1:**
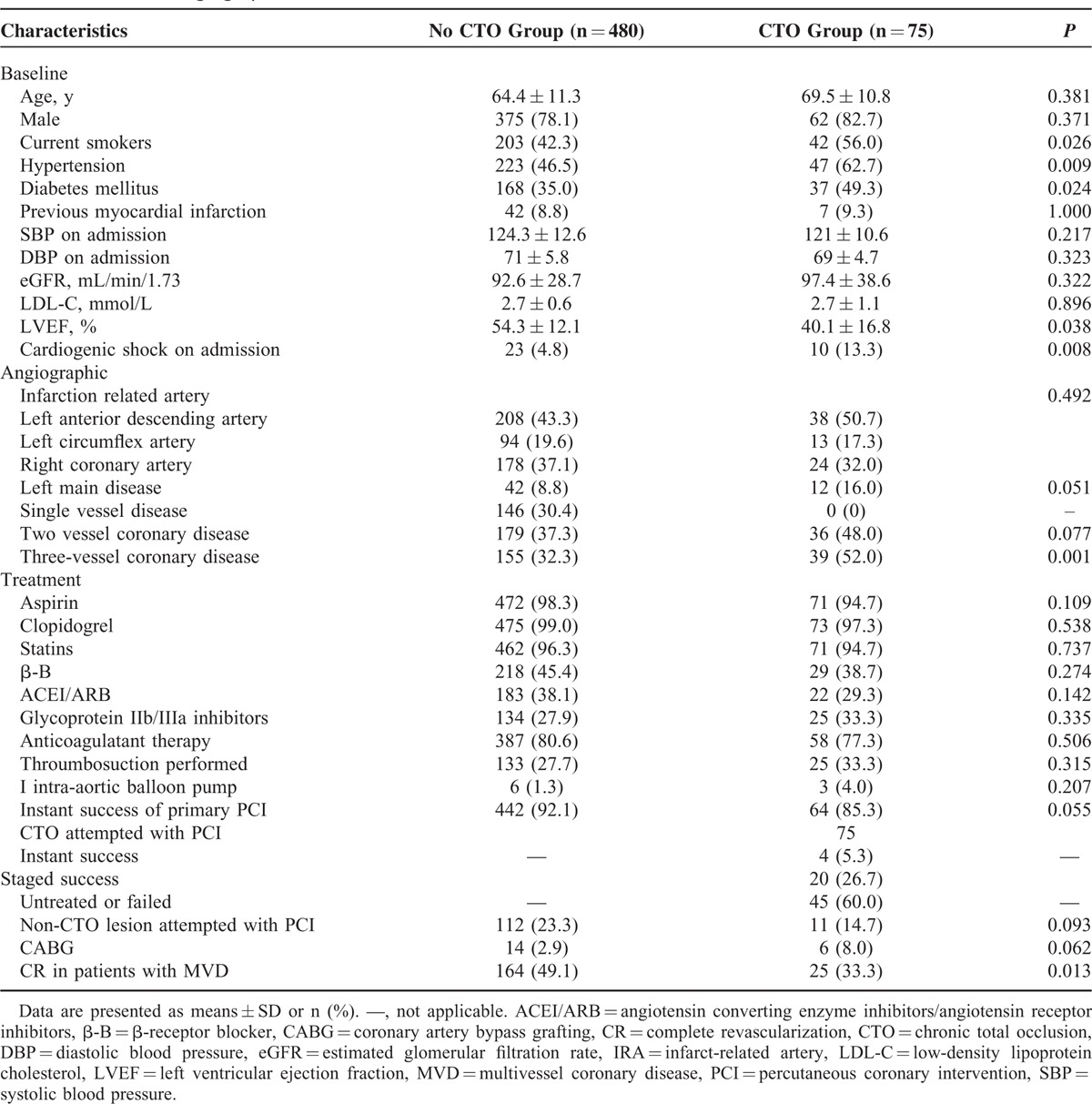
Baseline, Angiographic, and Treatment Characteristics

There were no significant differences with regarding to the distribution of IRAs between the 2 groups. Patients with CTO had a greater prevalence of 3-vessel coronary disease compared with patients without CTO (52.0% vs 32.3%, *P* = 0.001). Left main diseases were more observed in CTO group, the difference reached borderline significantly (16.0% vs 8.8%, *P* = 0.051). The instant success rate of primary PCI was 92.1% in no CTO group, 85.3% in CTO group (*P* = 0.055). Of 75 patients with CTO, for the reason of cardiogenic shock on admission, 4(5.3%) received instant additional PCI treating CTO after IRA being successfully recanalized, 26 patients underwent elective revascularization aimed at CTO including 20 staged PCI and 6 CABG. Most CTOs (60.0%) were left untreated or undergone PCI unsuccessfully. The percentage of patients received CABG in CTO group was 8.0%, 2.9% in no CTO group (*P* = 0.062). CR was achieved significantly lower in CTO group than in no CTO group (33.3% vs 49.1%, *P* = 0.013).

### Clinical Follow-up Within 1 Year

**FIGURE 1:**
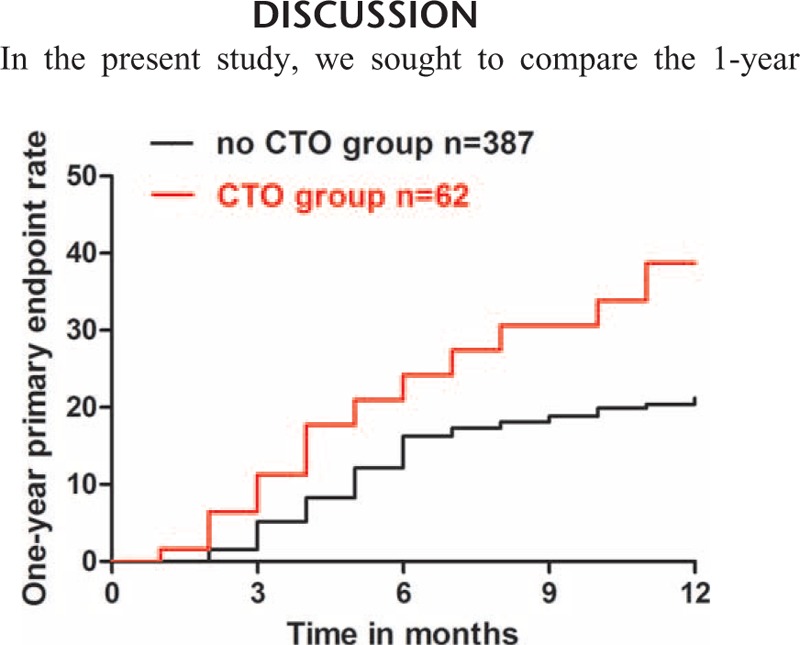
One-year primary endpoint rate in patients with STEMI underwent primary PCI. Patients with CTO versus patients without CTO, Log-rank *P* = 0.009. CTO = chronic total occlusion, PCI = percutaneous coronary intervention, STEMI = ST-elevation myocardial infarction.

The follow-up rate was 80.6% (387 patients) in no CTO group, 82.7% (62 patients) in CTO group. Detailed data concerning the clinical follow-up within 1 year are showed in Table 2. The 6-, 12-month combined occurrences of hospitalization from angina, reinfarction, heart failure, and re-revascularization was 11.3% versus 12.4% (*P* = 0.804) and 24.2% versus 15.0% (*P* = 0.068) for CTO group and no CTO group, respectively. The incidence of composite endpoint at 6-month in CTO group was insignificantly higher than in no CTO group (24.2% vs 16.5%, *P* = 0.142). The 12-month primary endpoint rate in CTO group was significantly higher than in no CTO group (38.7% vs 21.2%, *P* = 0.003) (Figure 1), mainly attributed to the difference of cardiac mortality.

**TABLE 2:**
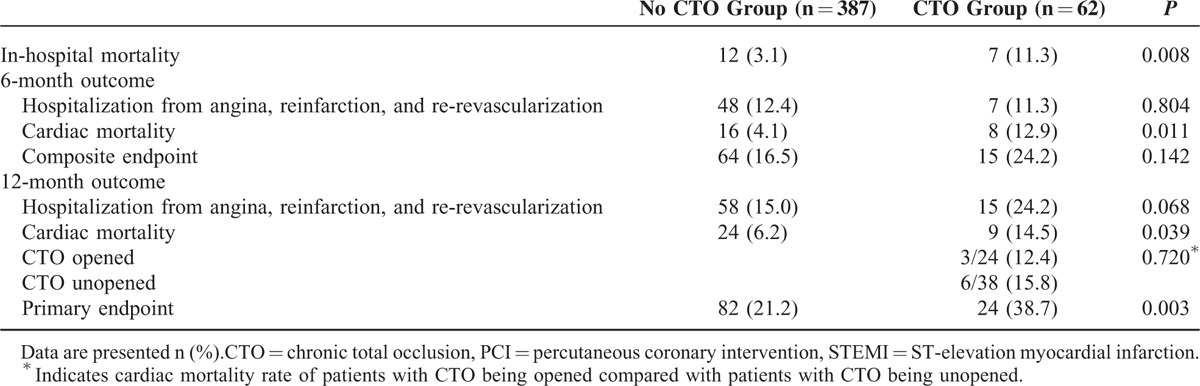
One-Year Follow-Up of Patients With STEMI Underwent Primary PCI

**FIGURE 2:**
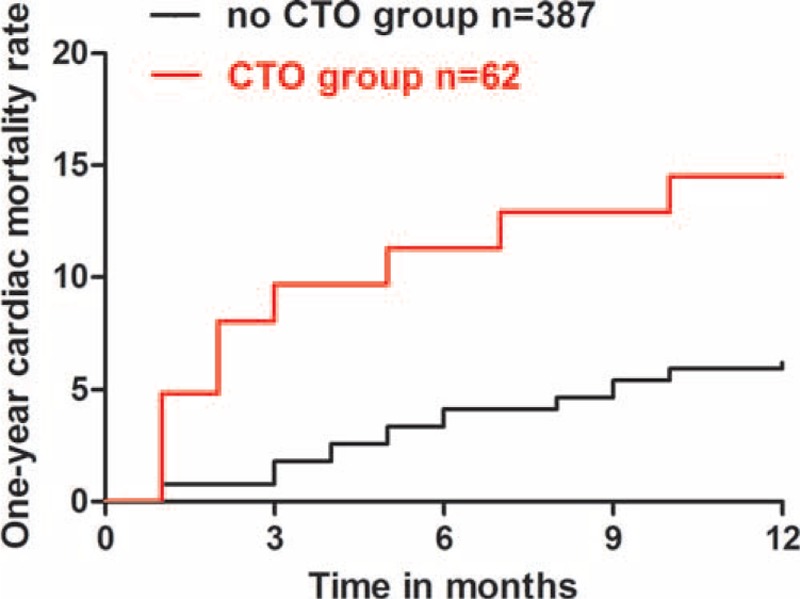
One-year cardiac mortality rate in patients with STEMI underwent primary PCI. Patients with CTO versus patients without CTO, Log-rank *P* = 0.016. CTO = chronic total occlusion, PCI = percutaneous coronary intervention, STEMI = ST-elevation myocardial infarction.

**FIGURE 3:**
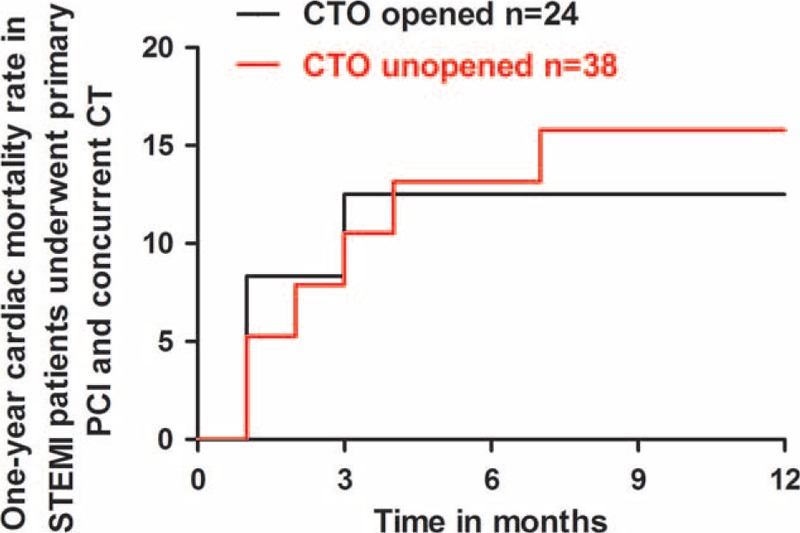
One-year cardiac mortality rate in STEMI patients underwent primary PCI and concurrent CTO. Patients with CTO being opened versus patients with CTO being unopened, Log-rank *P* = 0.744. CTO = chronic total occlusion, PCI = percutaneous coronary intervention, STEMI = ST-elevation myocardial infarction.

During hospitalization, 12(3.1%) patients died of cardiac reason in no CTO group, 7(11.3%) patients in CTO group (*P* < 0.05). The 7 died patients in CTO group included 2 patients who received instant additional PCI treating CTO after IRA being recanalized for the reason of cardiogenic shock on admission. The significant differences in the 6, 12-month cardiac mortality rate were maintained during 1 year follow-up, with 12.9% versus 4.1% (*P* = 0.011) and 14.5% versus 6.2% (*P* = 0.039) for CTO and no CTO groups, respectively (Figure 2). In CTO group, of 24 patients with CTO being opened, 3 (12.5%) died of cardiac reason during 12-month follow-up, this figure was 6(15.8%) of 38 patients with CTO being unopened, no survival difference was observed (*P* = 0.720) (Figure 3).

### Predictors of 1-Year Cardiac Mortality in Patients With STEMI Undergoing Primary PCI

To evaluate the independent predictors of 1-year cardiac mortality, multivariate Cox regression analysis was performed, as described above. All significantly different distributed clinical, angiographic, and procedure variables entered into Cox proportional hazards regression model were diabetes mellitus, cardiogenic shock on admission, 3-vessel coronary disease, and CTO in a non-IRA. Table 3 lists the predictors of 1-year cardiac mortality in patients with STEMI undergoing primary PCI. By multivariable analysis, the only 3 independent predictors of 1-year cardiac mortality were CTO in a non-IRA (hazard ratio: 4.183, 95% CI: 1.940–6.019; *P* = 0.001), cardiogenic shock on admission (hazard ratio: 3.286, 95% confidence interval: 1.097–9.845; *P* = 0.034), and 3-vessel coronary disease (hazard ratio: 2.678, 95% CI: 1.221–5.874; *P* = 0.014). After adjustment for possible confounders, diabetes mellitus alone (hazard ratio: 1.853, 95% CI: 0.876–3.782; *P* = 0.317) was not an independent predictor of 1-year cardiac mortality.

**TABLE 3:**
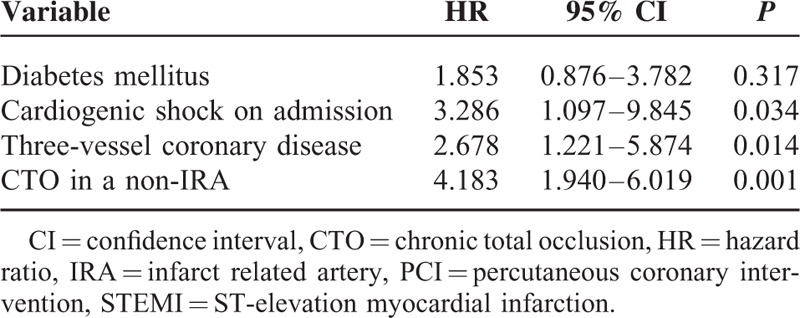
Predictors of 1-Year Cardiac Mortality in Patients With STEMI Undergoing Primary PCI

## DISCUSSION

In the present study, we sought to compare the 1-year outcomes of patients who underwent primary PCI for acute STEMI with and without CTO in a non-IRA and to investigate the impact of CTO on the prognosis during 1-year follow-up. The main findings of our investigation were following:

1. More than 1-8th (13.5%) of patients undergoing primary PCI for STEMI had CTO in a non-IRA, consistent with the incidence having been reported.^3,4^ CR was less achieved in CTO group than in no CTO group.
2. Patients with CTO in a non-IRA who underwent primary PCI for acute STEMI had a higher cardiac mortality.
3. After adjusted for differences in clinical, angiographic, and procedure variables, the presence of CTO in a non-IRA, cardiogenic shock on admission, and 3-vessel disease remained an independent predictor of greater 1-year cardiac mortality.

It has been reported that the presence of MVD is associated with poor clinical outcomes compared with single vessel coronary disease in patients with STEMI, mainly attributed to the increased mortality caused by heart failure.^3,11^ Although this situation was chiefly determined by the presence of CTO in non-IRAs.^12^ In our study, patients with STEMI and CTO who underwent primary PCI had a higher cardiac mortality during the 12-month follow-up period, suggested a poor prognosis. The reason concurrent CTO affects the prognosis adversely in patients with STEMI may be explained as follows. First, patients with STEMI and CTO have a greater risk profile which might influence clinical outcomes. In our study, patients with CTO in a non-IRA were more current smokers, had a greater prevalence of hypertension, diabetes compared with patients without CTO. Second, with the presence of CTO in a non-IRA, patients undergoing primary PCI more presented with a lower LVEF and cardiogenic shock on admission, compared with STEMI patients without CTO. In the setting of acute ischemia, STEMI patients with concomitant CTO were more revealed with a lower residual left ventricular function and lacking a compensation mechanism for the decreased LVEF. The preserved left ventricular function will be damaged obviously when suffers an AMI with a result of survival affected.^12^ Furthermore, the presence of CTO in a non-IRA would be associated with LVEF persistently decreasing during follow-up.^12^ The relationship between CTO in a non-IRA and increased occurrence of cardiogenic shock has been reported.^13^ It was reported that if cardiogenic shock on admission was present in patients with STEMI and CTO, then 1-year mortality increased by 1 to 4 times, 5-year mortality by 6 times compared with patients with STEMI without CTO.^3,4,14^ Third, revascularization was often performed incompletely in patients with STEMI and concurrent CTO in non-IRAs. Even if primary PCI was performed successfully, the ultimate CR including staged PCI and CABG might be affected attributed to extensive coronary disease, such as MVD with CTO or heavy calcified lesions, more being present. It was reported CR achieved by PCI was independently associated with survival benefit in patients undergoing primary PCI for STEMI and MVD.^5,6,15,16^ In the present study, CR was less achieved in CTO group than in no CTO group. Fourth, neurohormonal mechanisms in the progression of left ventricular remodeling may be involved in. The sympathetic activity adrenergic response was activated markedly in STEMI patients with certain prognostic biomarker upregulated, such as G protein-coupled receptor kinase 2 (GRK2).^17^ More patients with hemodynamic instability were observed in CTO group, the sympathetic activity adrenergic response was supposed to react more intensely in STEMI patients with CTO. Continuous activation of neurohormonal systems and adrenergic response were considered to be deleterious with persistent impact on left ventricular remodeling.^17^

Our study suggested CTO in a non-IRA, cardiogenic shock on admission, and 3-vessel disease might be an independent risk factor for greater 1-year cardiac mortality in patients with acute STEMI undergoing primary PCI. It was supposed that recanalization of CTO in non-IRAs to achieve CR may carry a beneficial effect.^18^ But in the present study, no survival difference was observed between patients with CTO being opened and unopened. There are several possible explanations concerning the unfavorable outcome. First, the CTO located in certain non-IRA and the range of viable territory may carry a different impact on prognosis.^19–21^ We did not evaluate whether the CTO vessels supplied a large area of myocardium, whether the viable myocardium subtended by the CTO vessel being present or not. In addition, most CTOs (60%) were left untreated in the present study. The sample size and the follow-up duration in our study may not be enough to address this question. Second, timing of subsequent PCI aimed at CTO after a successful primary PCI procedure may also play an important role related to prognosis. In general, CTO in a non-IRA was treated in an elective setting rather than in emergent circumstance. Current practice guidelines of primary PCI recommended urgent revascularization of diseased non-IRA vessels only in the presence of hemodynamic or electrical instability, it might carry a survival benefit, thus can be considerable.^14,22^ But the feasibility often deserves questionable. In the present study, owing to cardiogenic shock on admission, after culprit vessel being opened successfully, 4 patients with STEMI and concurrent CTO received CTO recanalized in the same procedure. However, 2 patients died of cardiovascular reason later during hospitalization. Additional PCI with CTO in a non-IRA in the prothrombotic milieu of the acute infarction phase might result in more adverse thrombotic events.^23^ Meantime, complicate PCI procedure will increase fluoroscopy exposure, increased contrast medium use will raise the occurrence of contrast induced nephropathy.^24^ In the ongoing EXPLORE trial, STEMI patients with CTO in a non-IRA undergoing primary PCI were randomized to staged PCI group aimed at CTO within 7 days after index procedure or standard medical treatment group. The results of EXPLORE trial will be helpful to answer the question whether elective percutaneous treatment of CTO within certain days after primary PCI improves LVEF and clinical outcomes of STEMI patients.^25^

## STUDY LIMITATIONS

The present study had several limitations. First, this was a single-center study in which only acute SETMI patients treated with primary PCI were enrolled. The patients with non-STEMI who underwent urgent PCI were not included. However, the objectives in our study reflected a representative population including a homogenous cohort during a 4-year period (2010–2013). Second, the strategy and technique on CTO in 1 single center might not adequately reflect the real world of treatment in patients with STEMI and concurrent CTO. Third, in the present study, CR by PCI and CABG was less achieved, in particular, most CTOs (45/75, 60.0%) were left untreated. The impact of CR on survival benefit might be analyzed inadequately. Fourth, we did not evaluate whether the viable myocardium subtended by the CTO vessel being present or not. So, the evidence supporting recanalization of CTO in non-IRAs might be not convincible. Fifth, the number of patients is relatively small, and the follow-up duration was only 1 year. Attributed to inadequate sample size, analyses of the diversity among the specified CTO coronary vessel on prognosis, timing of subsequent PCI aimed at CTO after a successful primary PCI procedure could not be carried out effectively. Much larger population and more prolonged follow-up are needed to investigate whether the hazardous effects of CTO in a non-IRA persist overtime in patients with STEMI receiving primary PCI.

## CONCLUSIONS

CTO in a non-IRA in patients with STEMI undergoing primary PCI is associated with a poor prognosis. The presence of CTO in a non-IRA, cardiogenic shock on admission, and 3-vessel disease might be an independent risk factor for greater 1-year cardiac mortality in patients with acute STEMI undergoing primary PCI. Prospective, multicenter, large-scale, and long-term follow-up designed study should be warranted in the future.
